# Delay-Period Activity and Executive Functions of the Prefrontal Cortex

**DOI:** 10.3390/brainsci10010003

**Published:** 2019-12-19

**Authors:** Aarron Phensy, Sven Kroener

**Affiliations:** School of Behavioral and Brain Sciences, The University of Texas at Dallas; 800 West Campbell Rd., Richardson, TX 75080, USA; ajp059000@utdallas.edu

The term “working memory” (WM) describes the ability to maintain and manipulate information in the memory for the guidance of goal-directed behavior. Working memory encompasses both storage and processing functions, and within cognitive psychology it represents an extension of an earlier concept, short-term memory, a limited-capacity temporary memory store, typified by the model proposed by Atkinson and Shiffrin [1]. The functions of WM range from the “simple” active retention of goal-related information to an import role in (speech)-comprehension, thinking, and planning in primates and humans [2]. Working memory is an important component of “cognitive control” or “executive functions” that are intricately associated with the prefrontal cortex (PFC). 

In the article [3], Shintaro Funahashi, who pioneered important behavioral and neurophysiological studies into the mechanisms of WM, provides an in-depth overview of how our understanding of working memory has developed from the abstract concepts originally proposed by Baddeley and Hitch [4] into discrete and tractable cellular mechanisms. Specifically, Funahashi highlights how neurons in the PFC maintain task-relevant information online during brief periods of time. Starting with work by Fuster, Goldman-Rakic, and others, neurophysiological studies in nonhuman primates have revealed that neurons in the PFC (and other cortical and thalamic areas that project to the PFC) show elevated levels of action potential firing during the memory phase of so-called delayed-response tasks, which are thought to be the neural correlate of temporary information storage in WM [5,6]. Delay-period activity may represent either retrospective information (memory about the properties of the cue) or prospective information (planning of the appropriate behavioral response). These alternative functions within WM can be dissected through the use of delayed-response tasks in which a subject is required to hold in mind the characteristics of a cue (e.g., location, color, texture) in order to guide behavior. Subpopulations of neurons in the PFC show a persistent and asynchronous delay period activity that is preferentially tuned to those stimulus features that are needed for the subsequent behavior. The rate and magnitude of tuning (specificity) certain PFC neurons can predict performance on these tasks. Other studies support the idea that this neural activity relates to the encoding of salient features and does not simply reflect premotor activity (prospective information). 

Another important concept is the idea that within working memory a control system of limited attentional capacity, termed the central executive by Baddeley and Hitch [4], determines the allocation of resources. Funahashi [3] describes recent research from dual task paradigms that found biological support for this idea. When subjects perform two simultaneous tasks, which both compete for working memory resources, electrophysiological recordings indicate significant overlap in PFC neurons that contribute to both tasks. By activating both populations of WM neurons simultaneously, one of the representations becomes attenuated, explaining the decreased performance on these tasks. Furthermore, because there is a correlation between task difficulty and level of attenuation this argues for a limited amount of resources that are available to maintain WM, and explains why the central executive can quickly become overwhelmed.

In addition to working memory, the executive functions of the PFC are important for the shifting of attention, the monitoring and updating of information, and the inhibition of prepotent responses [7]. A major role of the PFC during these behaviors is to monitor and update sensory information and memory processes both in itself, as well as in other cortical and subcortical brain regions. In humans, the ability to monitor one’s own memory processes is known as metacognition. In his review [3], Funahashi summarizes approaches to study the cellular mechanisms of this behavior in monkeys. To do so, experimenters first increased the difficulty of their tasks to cause errors and introduce a certain degree of uncertainty, then they allowed the animals to opt out of the trial for a reduced award. When animals chose to opt out of the trial this was interpreted to be an expression of doubt. Since doubt requires an introspective gauge of memory, this behavior can serve as a reasonable measure of metacognition in animals. Importantly, neuronal activity in PFC neurons during the delay period predicts whether or not the animal will opt out of the trial. 

In recent years considerable progress has been made in identifying the cellular elements that contribute to WM in mammals. Important aspects in this quest relate to the specificity of the input–output relationships (i.e., the functional heterogeneity of brain areas, and the laminar and synaptic organizations of these connections), the modulation of WM by neurotransmitters such as dopamine, and the physiological properties of the neurons involved [8]. However, an ongoing debate in the field centers on the question of what mechanisms give rise to the delay period activity, and whether descriptions of the persistent firing of specialized neurons are sufficient to understand the architecture of WM, or whether additional factors need to be considered. This debate is well exemplified by a recent “dual perspectives” series in the *Journal of Neuroscience* [9]. Constantinidis, Funahashi, Arnsten, and their colleagues present evidence for the viewpoint that persistent firing of PFC neurons represents information about stimuli held in the WM and that this activity determines WM performance. On the other hand, investigators around Earl Miller [10] argue that the persistence of delay period activity is largely an artifact of averaging activity across multiple trials, and that in contrast, activity in individual trials is sparse and occurs in bursts that may be related to oscillatory activity. While these investigators do not argue against the importance of delay period activity for WM, they suggest that dynamic changes in synaptic weights and the interactions between different rhythms in distinct cortical layers contribute significantly to delay activity. Thus, almost 50 years after the discovery of delay period activity in the PFC [6], important questions remain regarding the biological mechanisms of working memory, which are crucial for our understanding of higher cognitive function.
